# Three new genes associated with longevity in the European Bison

**DOI:** 10.1016/j.vas.2022.100266

**Published:** 2022-07-29

**Authors:** Evžen Korec, Lenka Ungrová, Jiří Hejnar, Adéla Grieblová, Kateřina Zelená

**Affiliations:** a Zoologická zahrada Tábor a.s., Dukelských Hrdinů 19, 170 00, Prague 7, Czech Republic; b Institute of Molecular Genetics of the Czech Academy of Sciences, Vídeňská 1083, 142 20, Prague 4, Czech Republic

**Keywords:** Longevity-associated genes, GWAS, European bison, Lifespan

## Abstract

Longevity-related genes have been found in humans, mice, dogs and in several other animal species. The goal of this study was to perform genetic analysis of long-lived European bisons with the aim to find genes that are associated with longevity using GWAS and further sequencing of a wider sample panel. European bison has a unique history of near extinction and the recovery of the species from just a few founder individuals. Together with the short medium lifespan, the expected genetic homogeneity makes bison a suitable model for studying longevity. Particular single nucleotide polymorphisms within three genes, *BCKDHB, FER1L6* and *SERPINI2*, were found significantly overrepresented in long-lived European bisons. In *SERPINI2*, the longevity-associated single nucleotide polymorphism localizes to an exon. In the protein encoded by the *SERPINI2* gene, amino acid leucine present in the reference European bisons is replaced by tryptophan in the long-lived animals. This study is the first to determine longevity-associated variants in genes in European bison. Association of the FER1L6 gene with longevity shows a possible sex dependency.

**Abbreviations**
L line: lowland line;
LC line: lowland-Caucasian line;
GWAS: Genome-wide association study;
SNP: single nucleotide polymorphism;
BCAA: branched-chain amino acids;
MSUD: maple syrup urine disease;
AFC: age at first calving

## 1 Introduction

European bison (*Bison bonasus*) was one of the key large herbivore species in European lowlands with a historic range across the whole Europe and East Asia. It became extinct in the wild at the beginning of the 20th century due to intensive unlimited hunting, poaching and habitat fragmentation (Pucek, Belousova, Krasiñska, Krasiñski & Olech, 2004). Only several dozen animals survived in zoos and private farms. In 1920, an attempt was made to restore the species from which two genetic lineages of European bison diversified. Although the lowland line (L) of European bison originated from only seven founders of *B. b. bonasus* subspecies, approximately 80% of the genes in the contemporary population came from as few as two founders. Thus, the average inbreeding coefficient in the L line is almost 50% (Tokarska, Pertoldi, Kowalczyk & Perzanowski, 2011). The *B. b. caucasicus* subspecies survives in hybrid form as the lowland-Caucasian line (LC), which originated from 12 animals and the inbreeding coefficient of the LC line is now 28% (Tokarska et al., 2011). The genus *Bison* has been studied from the evolutionary (Froese et al., 2017; Hassanin, An, Ropiquet, Nguyen & Couloux, 2013; Palacio et al., 2017), genetic (Gralak, Krasińska, Niemczewski, Krasiński & Żurkowski, 2004; Pertoldi et al., 2009; Tokarska et al., 2009b; Tokarska, Kawałko, Wójcik & Pertoldi, 2009a), behavioral (Gębczyńska, Gębczyński & Martynowicz, 1991; Lott, 1991) and conservational (Gates, Freese, Gogan & Kotzman, 2010; Olech, 2008;) points of view. The European bison is an exceptional genetic model of a large mammal with a high degree of inbreeding.

In our previous study we determined the median lifespan of the European bison as only 3.54 years. The median lifespan of females (6.01 years) exceeded more than twice the median lifespan of males (2.71 years). This sex-related difference in longevity is the biggest one so far described in mammals (Korec et al., 2019). The low median lifespan of European bison is caused by an enormous mortality rate of newborn and young calves. Up to 50% of calves die on the day of birth and 11% - 44% of calves die in the first year of life. Mortality rate is overall higher in males (Urošević, Dameski, Mandić & Stanišić, 2022). Higher mortality of European bison males was previously described in the population from Białowieża forest, Poland. Posthitis which affects only males was one of the common causes of death together with injuries caused by other bison (Pucek, Belousova, Krasińska, Krasiński & Olech, 2002). Male bisons are prone to be more aggressive towards each other than females. Older and larger males are more aggressive towards younger ones as shown in the closely related American bison (King, Caven, Leung, Ranglack & Arcilla, 2019) which can lead to earlier death caused by trauma.

Despite the low median lifespan, some European bisons live up to 28 years (Pucek et al., 2002; Weigl, 2005). Both these facts together with a high degree of inbreeding make the European bison an interesting model for studying longevity. Since reintroduction of this species into the wild is continuing, the genetic background of this unusual lifespan distribution with some individuals exceeding almost eight times the median will be also important for creating stable and viable herds.

Longevity-associated genes have been described in humans, mice, dogs and in other animals (Korec, Ungrová, Hejnar & Grieblová, 2022; Pilling et al., 2017; Shadyab et al., 2015; Singh, Demmitt, Nath & Brunet, 2019). In cattle, longevity is most of the time described as a productive lifespan, not a whole-life lifespan (Mészáros, Eaglen, Waldmann & Sölkner, 2014; Steri, Moioli, Catillo, Galli & Buttazzoni, 2018). Only one study so far focuses on whole-life lifespan in cattle (Zhang et al., 2021). No genes associated with longevity have been described in European bison yet.

The aim of this study was to identify longevity-associated genes in the European bison using genome-wide association study (GWAS) and further sequencing of a wider sample panel.

## 2 Methods

### 2.1 Sampling

Hair samples with roots from European bison individuals were obtained from various breeders during the years 2016–2020. Samples were divided into two groups defined by the age of the examined bisons. The group of long-lived bisons contained samples from individuals older than 14 years, which were considered as long-lived according to the previous study on the European bison longevity (Korec et al., 2019). For the reference group, we sampled bisons aged between 3 and 5 years. European bison in the reference group aged 3–5 years are less than 5% likely to live up to 14 years. This was found during research aiming to determine the median lifespan of European bison (Korec et al., 2019). All animals in the reference group were selected randomly. Overall, 22 samples of long-lived bisons and 20 samples of reference bisons were used for this study (Table A1 in appendix). Since the reference group could contain the 5% of long–living individuals, monitoring of this group will continue to refine our results afterwards.

### 2.2 DNA isolation, SNP genotyping

DNA was isolated from hair samples using a Qiagen DNeasy Blood & Tissue Kit and the standard phenol–chloroform DNA isolation protocol. DNA was eluted in 20 μl to 100 μl elution solution. The concentration and purity of isolated DNA was checked using a spectrophotometer. The required length of 5000 base pairs for single nucleotide polymorphism (SNP) genotyping was checked in 2% agarose gel. Suitable samples were diluted or concentrated by ethanol precipitation to the required concentration of DNA for 20–30 ng/μl. Overall, 27 samples (11 long-lived and 16 reference) were genotyped using the SNP array. According to the previous successful genotyping of bison individuals using bovine SNP chips (Oleński et al., 2020; Oleński, Kamiński, Tokarska & Hering, 2017; Stronen et al., 2018), we decided to genotype our samples using Illumina BovineHD BeadChip at Neogen laboratory, 4131 N. 48th St. Lincoln, NE 68504, USA. This chip allows analysis of more than 770,000 SNPs.

### 2.3 GWAS

Statistical analysis and the necessary steps preceding association analysis and association analysis itself were performed using PLINK v1.90b6.16 (Purcell et al., 2007). We checked our results according to commonly used quality parameters that were adjusted to fit our data (Marees et al., 2018). SNPs that were missing in more than 1% (–geno 0.01) of the samples were excluded from further analysis. All samples used for further analysis had more than 95% of SNP markers genotyped (–mind 0.05). In total, 546,352 variants from 27 bisons passed the primary data cleaning. Further, SNPs with minor allele frequencies lower than 5% (–maf 0.05) were also excluded from the association analysis. At the end, 13,080 SNP variants from 27 bisons passed for the final genome-wide association analysis (GWAS). Case/control association and standard 1df chi-square allelic test was used for GWAS (–assoc), without considering sex and genetic line of the sampled individuals. This test was used as a prediction tool for finding candidate SNPs. Considering the low number of samples used for GWAS, deeper stratification of the dataset with more phenotype information would result in even less reliable results. SNPs from the analysis were then further sequenced in a wider sample panel to verify predicted associations with longevity. *P*-values were adjusted using Benjamini-Hochberg correction (Benjamini & Hochberg, 1995) and Bonferroni correction. Principle component analysis (PCA) vectors and values were also exported using PLINK.

For each individual, inbreeding coefficient was calculated using expected and observed homozygosity (–het) from the dataset of 546,352 variants. Individuals were divided into the two genetic lines for this analysis. The analysis was also performed in PLINK.

PCA, quantile-quantile (QQ), and Manhattan plots for visualization of association analysis were constructed in R Studio (RStudio Team, 2020) using packages ggplot2 (Wickham, 2016), lattice (Sarkar, 2008) and qqman (Turner, 2018).

### 2.4 Sequencing

According to the result of GWAS, genomic position of all candidate SNPs was checked in the *Bos taurus* reference genome. Since the Illumina BovineHD BeadChip was designed using older assembly UMD 3.1.1 we used Liftover tool (https://github.com/sritchie73/liftOverPlink) to obtain genomic positions of the SNPs in the newer ARS-UCD 1.2 genome assembly. Three candidate SNPs with the lowest P-value according to GWAS localized in introns of three genes and one candidate SNP localized in an exon of annotated gene were PCR amplified and sequenced in 40 samples including those used for GWAS (two samples were not successfully sequenced in any of the selected genes). DNA samples were sequenced by SEQme s.r.o., 26,301 Dobris, Czech Republic. PCR condition were as follows: after the initial denaturation at 95 °C for 120 s, 33 cycles of 20 s at 95 °C, 30 s at 59 °C, and 120 s at 65 °C were performed and followed by final extension of 300 s at 65°The primers used for PCR amplification are available in Appendix (Table A2).

Statistical significance of the SNP genotype distribution within the long-lived and reference group of all sequenced bisons was tested in R Studio (RStudio Team, 2020) using Fisher's exact test (Fisher, 1934). The same test was also used to determine whether there is any significant difference in SNP genotype distribution between males and females. Individuals from the reference group that had the longevity associated genotype were also used in this test as they could potentially be long-living as well.

All analyses and plots were performed using R programming language version 4.1.3 (R Core Team, 2022).

## 3 Results

### 3.1 GWAS

**Fig. 1:**
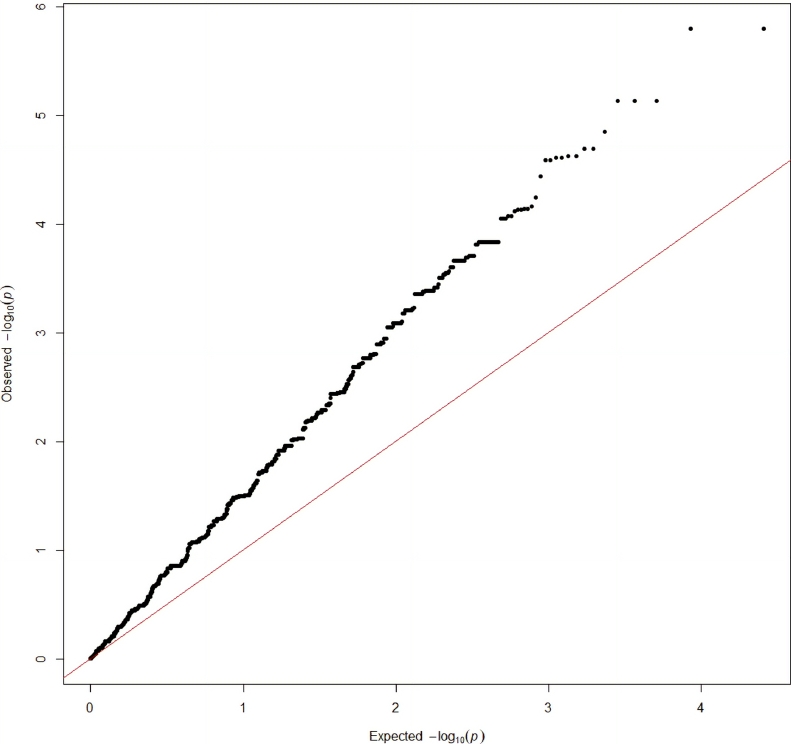
QQ plot of the association analysis after data cleaning. Red line represents the expected *P*-value, black dots represent the observed *P*-value from the association analysis. (For interpretation of the references to colour in this figure legend, the reader is referred to the web version of this article.)

**Fig. A1:**
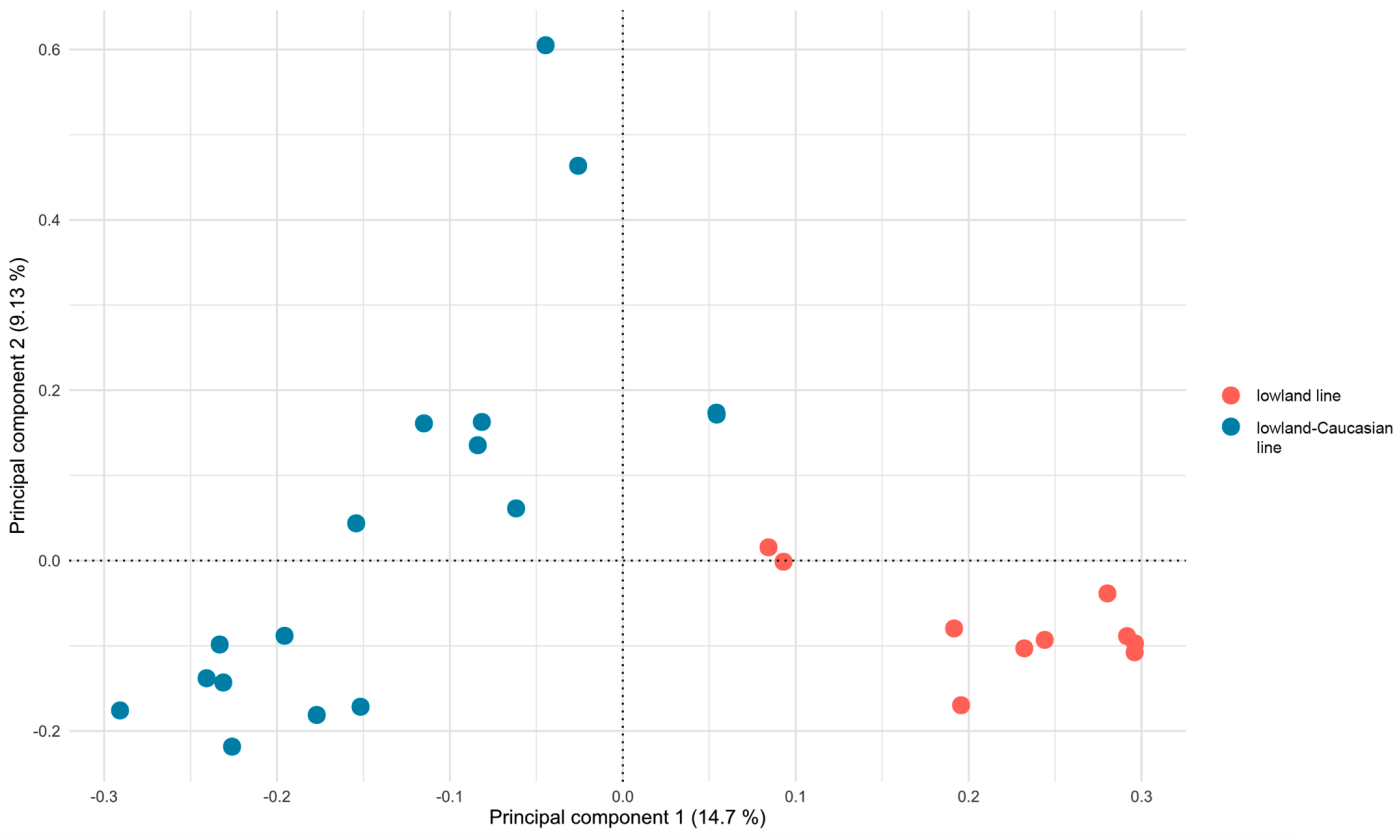
PCA plot of European bison individuals. Figure shows that there is a population stratification due to presence of two lineages of European bison in the dataset, the lowland line and the lowland-Caucasian line. Orange dots represent lowland line. Turqoise dots represent lowland-Caucasian line. (For interpretation of the references to colour in this figure legend, the reader is referred to the web version of this article.)

**Fig. A2:**
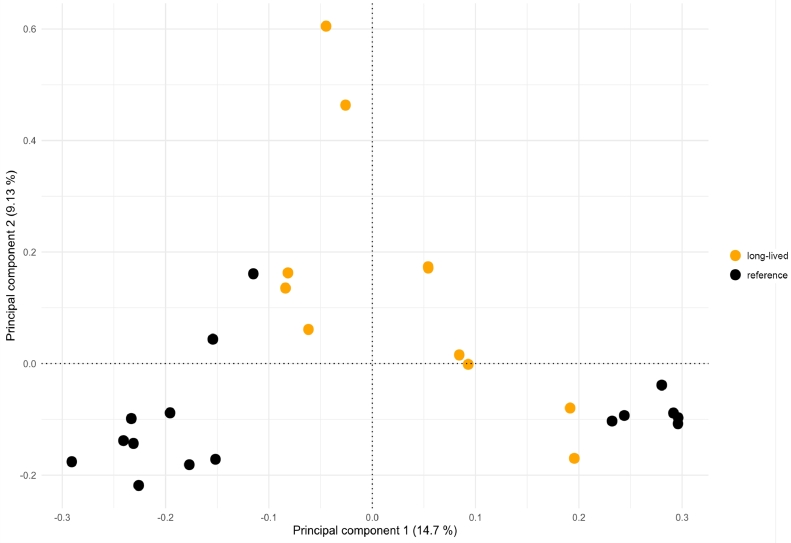
PCA plot of European bison individuals showing distribution of the samples of long-lived animals in the dataset of two lineages (Fig A1). Orange dots represent long-lived individuals. Black dots represent reference individuals. (For interpretation of the references to colour in this figure legend, the reader is referred to the web version of this article.)

QQ plot depicting a clear difference between the observed and expected P-values after accounting for population structure is presented in Fig. 1. Genomic inflation estimation (λ) = 1.94 suggests population stratification as well as the PCA plot (Fig. A1 in appendix) that shows a clear differentiation of two European bison lines presented in the dataset. However, the distribution of long-lived animals within the two lines is equal (Fig. A2 in appendix). Average inbreeding coefficient based of homozygosity in LC line individuals was -0.072, in L line 0.185.

**Fig. 2:**
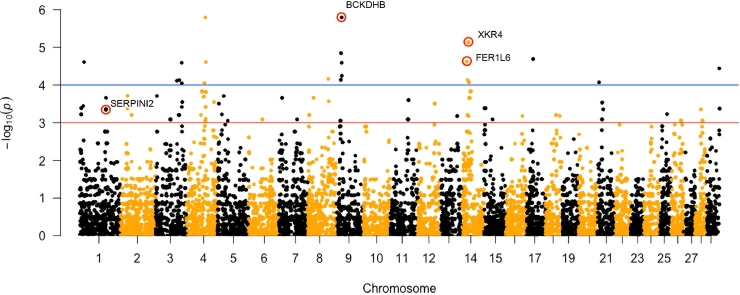
Manhattan plot of the GWAS results. The blue line represents a significance threshold of 1.0e-04. The red line represents a significance threshold of 1.0e-03. SNPs chosen for further analyses are circled. (For interpretation of the references to colour in this figure legend, the reader is referred to the web version of this article.)

In total, 23 SNPs passed the significance threshold (1.0e-04) which was set as the lowest *P*-value for the screening of SNPs localized in introns. Out of this, seven SNPs were located in genes annotated in the *Bos taurus* reference genome. According to the results of GWAS, we selected three SNPs located in the intron region with the lowest *P*-value and high genomic coverage for further sequencing (Fig. 2). In addition, one SNP localized in the exon region with the lowest *P*-value was also analyzed as a candidate longevity-related locus by sequencing in the whole sample panel (Fig. 2). Results from the association analysis for the four selected SNPs and their positions in particular genes are shown in Table 1.

**Table 1:** Table of the GWAS results for four candidate SNPs sorted by the lowest P-value. P-value, unadjusted P-value; P-value_BH, P-value adjusted by Benjamini-Hochberg correction; P-value_B, P-value adjusted by Bonferroni correction; CHR, chromosome; SNP, single nucleotide polymorphism; BP, genomic position (base-pair) in ARS-UCD 1.2 genome; A1, minor allele; F_A, frequency of minor allele in long-lived group; F_U, frequency of minor allele in reference group; A2, major allele; CHISQ, basic allelic chi-square test; OR, estimated odds ratio; NA, not applicable.

Gene	*P*-value	*P*-value_BH	*P*-value_B	Position	CHR	SNP	BP	A1	F_A	F_U	A2	CHISQ	OR
*BCKDHB*	*1.61E-06*	*0.0105*	*0.021*	*intron*	*9*	*BovineHD0900005530*	*20,052,757*	*T*	*0.7727*	*0.125*	*G*	*23.02*	*23.8*
*XKR4*	*7.38E-06*	*0.01928*	*0.09639*	*intron*	*14*	*BovineHD1400007066*	*22,706,112*	*G*	*0.5*	*0*	*A*	*20.09*	*NA*
*FER1L6*	*2.39E-05*	*0.02399*	*0.3121*	*intron*	*14*	*ARS-BFGL-NGS-82,859*	*15,929,822*	*T*	*0.4545*	*0*	*C*	*17.85*	*NA*
*SERPINI2*	*4.45E-04*	*0.06052*	*1*	*exon*	*1*	*BovineHD0100047129*	*99,998,320*	*G*	*0.4091*	*0.0313*	*T*	*12.33*	*21.5*

### 3.2 Sequencing

**Fig. 3:**
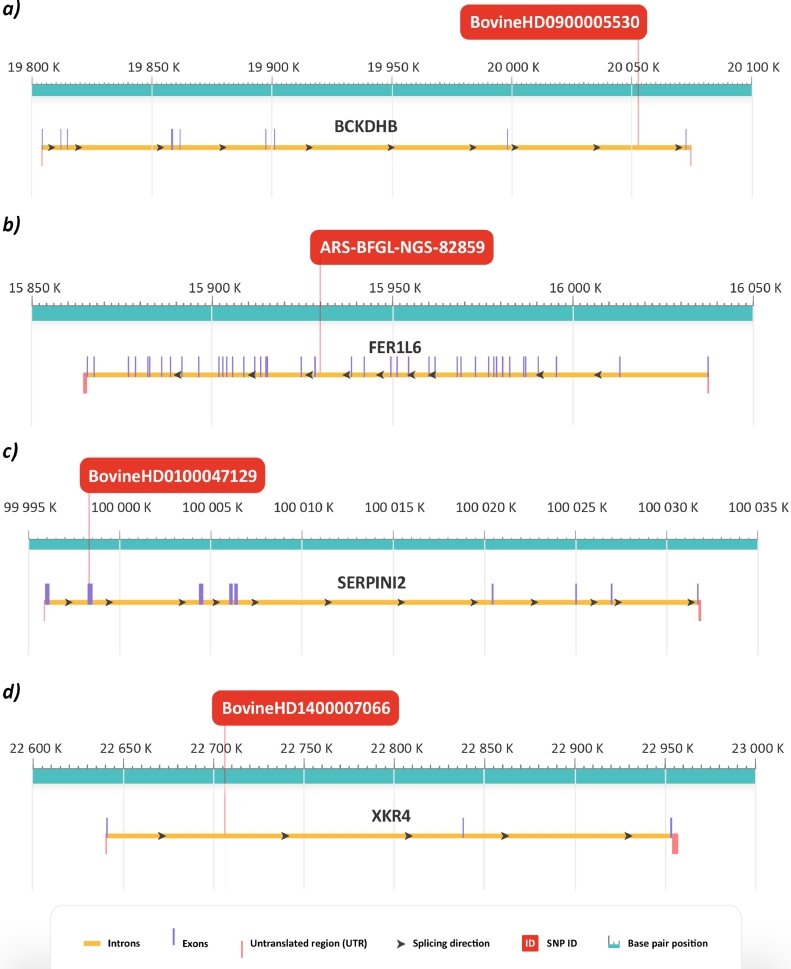
Genomic region of (a) chromosome 9 showing gene *BCKDHB* with SNP associated with longevity; (b) chromosome 14 showing gene *FER1L6* with SNP associated with longevity; (c) chromosome 1 showing gene *SERPINI2* with SNP associated with longevity; (d) chromosome 14 showing gene *XKR4*.

**Fig. 4:**
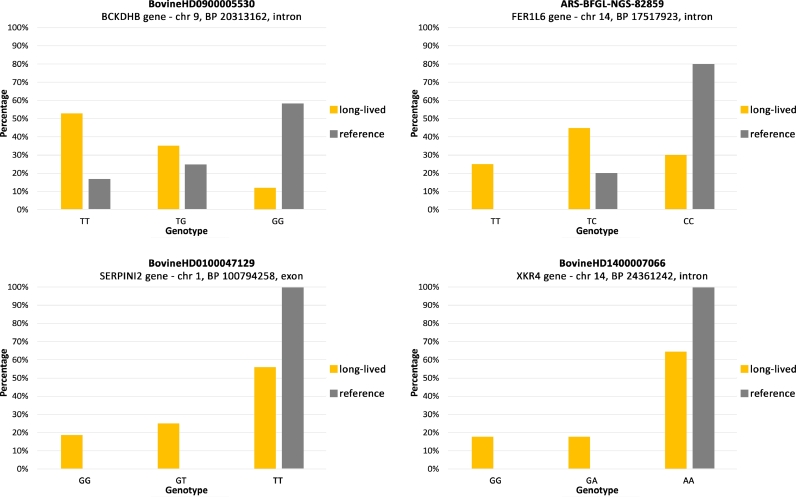
Distribution of genotypes in SNPs of investigated genes. Orange columns = long-lived group, black columns = reference group. (For interpretation of the references to colour in this figure legend, the reader is referred to the web version of this article.)

For the SNP BovineHD0900005530, most significantly longevity-associated according to the GWAS results, which localized to an intron of the *BCKDHB* gene (Fig. 3Fig. 3.(a)), association of nucleotide T with longevity in both homozygous and heterozygous form was significant according to Fisher's exact test (*P*-value = 0.02922) after sequencing of the whole sample panel. Genotype TT was found in 53% of the long-lived bisons and only in 17% of reference samples. Thirty five percent of the long-lived bisons were heterozygous with genotype TG in comparison to 25% heterozygous from the reference group. Only 12% of the long-lived bisons had genotype GG compared to 58% of the reference group animals (Fig. 4).

For SNP ARS-BFGL-NGS-82,859 localized in an intron of the *FER1L6* gene (Fig. 3bFig. 3. (b)), association of allele T with longevity in both homozygous and heterozygous forms was significant according to Fisher's exact test (*P*-value = 0.009786) after sequencing of the whole sample panel. Twenty five percent of the long-lived bisons had the homozygous genotype TT which has not been found in the reference group. The heterozygous genotype TC was found in 45% of the long-lived bisons and in 20% of the reference bisons. Genotype CC was present in 30% of the long-lived bisons and in 80% of the reference bisons (Fig. 4).

**Fig. A3:**
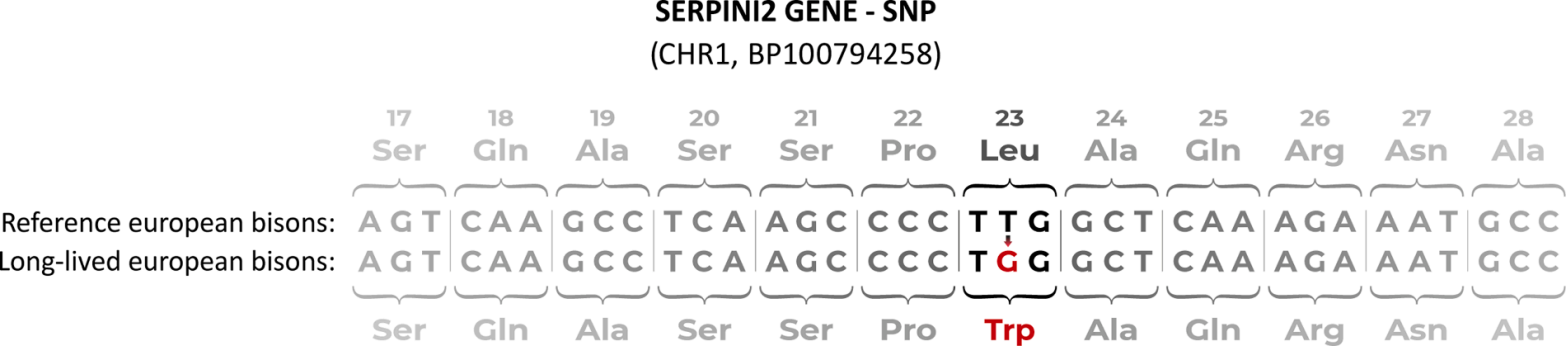
Nucleotide substitution and amino acid change in SNP BovineHD0100047129 located in the SERPINI2 gene.

In the second exon of the *SERPINI2* gene in SNP BovineHD0100047129 (Fig. 3cFig. 3. (c)), the allele G is significantly associated with longevity in both homozygous and heterozygous forms according to Fisher's exact test (*P*-value = 0.0391) after sequencing of the whole sample panel. Nineteen percent of the long-lived group had the homozygous genotype GG, which was not present in individuals from the reference group. The heterozygous genotype GT was found in 25% of the long-lived bisons. Again, this genotype has not been detected in individuals from the reference group. Homozygous genotype TT was found in 56% of the long-lived group and in 100% of the reference group (Fig. 4). With this nucleotide substitution T → G, amino acid leucine present in the reference group was replaced by tryptophan in the long-lived group (23 Leu → 23 Trp; Fig. A3 in appendix).

Even though SNP BovineHD1400007066 located in an intron of the *XKR4* gene (Fig. 3dFig. 3. (d)) had a low *P*-value in GWAS, association of allele G with longevity turned out not significant according to Fisher's exact test (*P*-value = 0.1262) after sequencing of the whole sample panel. Both homozygous genotype GG and heterozygous genotype GA containing the candidate longevity-associated allele were found in 17% of the long-lived group, but not in individuals from the reference group. Homozygous genotype AA was found in 66% of the long-lived group and in 100% of the reference group (Fig. 4).

### 3.3 Sex differences

**Fig. 5:**
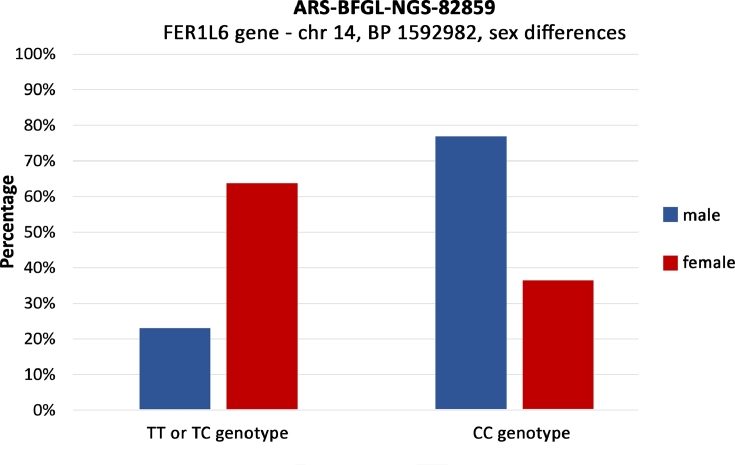
Difference in distribution of genotypes in SNP of the FER1L6 gene between male and female bisons.

Long-lived females were not significantly overrepresented in longevity-related allele carriers when all three loci (*FER1L6, BCKDHB* and *SERPINI2*) were considered together. Looking at the *FER1L6* gene separately, the longevity-associated allele in both homozygous and heterozygous form was significantly overrepresented in females (*P* = 0.03542, Fig. 5). Also, all long-lived sampled females had the longevity-associated genotypes in BCKDHB gene, but there was no significant overrepresentation of those genotypes in females compared to males. There was no significant difference in genotype representation between female and male bisons in the SERPINI2 gene.

## 4 Discussion

European bison is a unique species of large mammal with very high inbreeding coefficient, which reaches 50% in the L line and 28% in the LC line (Tokarska et al., 2011). The high inbreeding coefficient is due to the fact that the rescue of the species was carried out by crossing a very small number of founder animals. Despite the expected genetic uniformity, the lifespan of individual animals is highly variable. In our study, average inbreeding coefficient per sample was -0,072 for LC line and 0,185 for L line. Lower coefficients can be results from selective breeding of non-related individuals and also of small size of the dataset used in this study. Also, different methods of calculating the inbreeding coefficient can lead to different results (Li, Strandén, Tiirikka, Sevón-Aimonen & Kantanen, 2011).

Although the median lifespan is only 3.54 years (Korec et al., 2019), some animals live to 28 years (Pucek et al., 2002; Weigl, 2005). Very interesting is the fact, that the median lifespan of females (6.01 years) exceeded more than twice the median lifespan of males (2.71 years). The genus *Bison* has the biggest sex-related difference in longevity among mammals. Statistical evaluation of the lifespan of individual animals does not correspond with the normal distribution that is common for most animal species (Korec et al., 2019).

The European bison thus represents a very interesting model for the study of longevity-associated genes. Identifying genes associated with longevity in European bison could be useful for long-term conservation of this species and could improve current and future reintroduction programs thanks to selective breeding and deeper knowledge about its genetic background.

We used GWAS as a tool to predict candidate longevity-related SNPs in European bison. We did not divide our samples in the two genetic lines for the analysis since the association of the genes and their variants with longevity is not always specific for certain subpopulation of one species and can be found also in different species. For example, associations of SNPs in *MC2R* gene found in human (Pilling et al., 2017) and also in a dog (Korec et al., 2022). *FOXO3* also plays a role in longevity in multiple species (Sanese, Forte, Disciglio, Grossi & Simone, 2019). We decided to use more relaxed parameters for the results of the GWAS because confirmatory sequencing on larger sample panel followed the analysis. Non-significant results of Bonferroni correction could be caused by its strictness when it is used on a small sample size (Hinrichs, Larkin & Suarez, 2009; Kuo, 2017). Therefore, we followed the results of Benjamini-Hochberg correction in accordance with van den Berg, Vandenplas, van Eeuwijk, Lopes & Veerkamp, 2019 and also because GWAS was applied just as a primary screen in our research.

Even though the results from the GWAS had weaker power because of the small sample size, we were able to verify the findings with further sequencing and statistics in a larger sample panel.

Thanks to this strategy we were able to perform a case/control association study on a species where it is very complicated to collect enough samples for a GWAS identification of candidate SNPs. Since individuals that were used as a reference group could be possibly long-living, monitoring of these animals will continue.

Previously, we succeeded with similar strategy in finding longevity-related SNPs in the genome of purebred dog Cane corso (Korec et al., 2022). Using this methodology, we have now identified three SNPs that are significantly overrepresented in long-lived individuals of European bison.

One of the significant SNPs is located in an intron of *BCKDHB* gene which encodes the E1 beta subunit of the branched-chain keto acid dehydrogenase, which is a multienzyme complex associated with the inner membrane of mitochondria. This enzyme complex is active in the catabolism of branched-chain amino acids. Mutations of this gene have been associated with the maple syrup urine disease (MSUD) type 1B, a disease characterized by a maple syrup odor of the urine, mental and physical retardation, feeding problems and dihydrolipoamide dehydrogenase deficiency (Wang, Qi, Li & Zhao, 2012). This disease can affect cattle (Harper, Dennis, Healy & Brown, 1989). It was shown that MSUD can cause DNA damage (Scaini et al., 2012) that can have a direct influence on longevity (Freitas & de Magalhaes, 2011). Considering these facts, BCKDHB could play an important role in longevity, but further research would be needed to prove this hypothesis. The association of the BCKDHB gene with longevity has not been described so far.

Second statistically significant SNP is located in an intron of the *FER1L6* gene (FER-1 like family member 6) which is associated with diseases including cerebellar ataxia type 43 (Kanuka et al., 2020) and Miyoshi muscular dystrophy (Bansal & Campbell, 2004). The association of the *FER1L6* gene with longevity has not been described so far. If we consider the overrepresentation of the longevity-associated allele in European bison females, FER1L6 was previously described as a factor influencing age at first calving (AFC) in cattle (Mota et al., 2017). AFC could also influence not just productive lifespan but also longevity of the cows (Valchev, Marinov & Angelova, 2020). It was previously described in humans that longevity associated SNPs could be sex-dependent (Zeng, Nie, Min, Chen & Liu, 2018). Our finding supports the results of previous studies considering sex-dependent longevity in European bison based on mortality rates and pedigree analysis (Korec et al., 2019; Urošević et al., 2022), However these studies discuss their findings considering factors that disadvantage males from reaching longer lifespan such as sex-specific diseases and behavioral differences. Conversly our results show a possible advantage in European bison females compared to males on a genetic and probably physiological level regarding longevity. However, more in-depth research would be needed to prove the importance of the SNP in *FER1L6* gene in European bison females regarding sex-dependent longevity.

We also described one SNP associated with longevity that is localized in an exon of the *SERPINI2* gene. Nucleotide substitution in the SNP position changes amino acid leucine, which is present in the reference bisons, to tryptophan present in the long-lived bisons. Structural changes of the protein coded by the SERPINI2 gene produced in the long-lived bisons will be the subject of our future research.

The *SERPINI2* gene (SERPIN family I member 2) encodes a member of a family of proteins that acts as an inhibitor of serine protease. These proteins act in the regulation of a variety of physiological processes including coagulation, fibrinolysis, development, malignancy and inflammation (Law et al., 2006). Expression of the encoded protein is downregulated in pancreatic and breast cancer and it is associated with acinar cell apoptosis and pancreatic insufficiency when absent in mice (Higgins, Grehan, Wynne & Worrall, 2017). *SERPINI2* deficient mice are growth retarded, have abnormal immunity and reduced lifespan (Loftus et al., 2005). Association of the SERPINI2 gene with lifespan that was also found in the mice may suggest that the association of this gene with longevity could be species independent.

This study has its limits due to the low number of sampled European bisons. Also, the necessity to align our GWAS data to the reference genome of domestic cattle could have reduced our chance to identify some candidate SNPs. It will be useful to confirm these results in a study with a larger number of samples and refine the statistical analysis in the future when it will be clear what animals in the reference group will score as long-lived. Also, more in-depth analysis including phenotypic information such as sex and genetic line on large sample panel could bring more insight into this topic. This is a plan for our future research. However, even the limited size of the current sample panel allowed statistical analyses to be performed and determine the statistical significance of our findings.

## 5 Conclusions

Three genes, *BCKDHB, FER1L6* and *SERPINI2*, were newly identified to be associated with longevity in European bisons using GWAS and DNA sequencing.

In *BCKDHB* and *FER1L6* genes, the longevity-associated SNPs are localized in introns. Association of the *FER1L6* gene also shows a possible sex-dependency.

In the *SERPINI2* gene, the longevity-associated SNP is localized in an exon. Amino acid leucine present in the protein coded by the *SERPINI2* gene in reference European bisons is replaced by tryptophan in the long-lived European bisons.

## Funding

This research received no external funding.

## Author contributions

All authors have read and agreed to the published version of the manuscript.

## Availability of data and materials

The datasets used in this study are available from https://www.ebi.ac.uk/eva/?Study-Browser&browserType=sgv accession number: PRJEB51724.

## Declarations

### Ethics approval and consent to participate

All samples were obtained non-invasively. Owners of the European bisons collected and provided all samples. Bison hair samples were collected not directly for this study. The hair samples were collected for the needs of the owners of the animals, only then the owners sent the samples to be used for this study. All owners approved the experimental protocols beforehand and all methods were performed in accordance with the relevant guidelines and regulations. Informed consent and permission to use the provided samples in this study was obtained from all owners.

### Consent for publication

Not applicable.

## CRediT authorship contribution statement

**Evžen Korec:** Conceptualization, Project administration, Supervision, Validation, Writing – original draft. **Lenka Ungrová:** Methodology, Visualization, Writing – original draft. **Jiří Hejnar:** Methodology, Supervision, Validation, Writing – review & editing. **Adéla Grieblová:** Methodology. **Kateřina Zelená:** Writing – review & editing.

## Declaration of Competing Interest

The authors declare that they have no known competing financial interests or personal relationships that could have appeared to influence the work reported in this paper.

## Figures and Tables

**Table A1:** Dataset of samples used for the research. Samples used for SNP genotyping are in bold. LC = lowland-Caucasian line, L = lowland line.

Name	Group	Lineage	Sex	Date of birth	Institution
Bärnburger	Long-lived	LC	M	02.07.2003	Hirschfeld ZOO
Baron	Long-lived	L	M	01.01.2001	Minsk ZOO
Bransk	Long-lived	LC	M	02.06.1997	The Aspinall Foundation
Donna	Long-lived	L	F	29.06.2002	Fota Wildlife Park
Elvis	Long-lived	L	M	01.10.2003	Wilhelma ZOO
Erika	Long-lived	L	F	15.05.2001	Fota Wildlife Park
Gama	Long-lived	LC	F	29.08.1999	Tallin ZOO
Indi	Long-lived	LC	F	09.05.2006	Wisentgehege Springe
Izafatty	Long-lived	LC	F	31.08.2003	The Aspinall Foundation
Izvara	Long-lived	LC	F	06.07.2000	The Aspinall Foundation
Maika	Long-lived	LC	F	11.10.2000	Tallin ZOO
Nina	Long-lived	L	F	21.06.2002	Fota Wildlife Park
NL-25*	Long-lived	LC	M	14.06.1998	Roev Ruchey ZOO Krasnoyarsk
Plucik	Long-lived	L	M	01.09.2001	Duisburg ZOO
Postójka	Long-lived	L	F	01.01.2002	Bydgoszcz ZOO
Pristina	Long-lived	LC	F	25.07.1995	Prague ZOO
Raisa	Long-lived	LC	F	16.06.2000	Tallin ZOO
Rimpsa	Long-lived	LC	F	31.05.2000	Tallin ZOO
Sphinx	Long-lived	LC	F	21.05.1998	Wisentgehege Springe
Splunkunyr	Long-lived	LC	M	06.05.2005	Wisentgehege Springe
Thira	Long-lived	LC	F	01.04.1998	Hirschfeld ZOO
Zwursel	Long-lived	LC	F	10.05.2002	Hirschfeld ZOO
Curt	Reference	LC	M	20.06.2017	Wisentgehege Springe
Diestera	Reference	L	F	18.06.2018	Wisentgehege Springe
Deisterix	Reference	L	M	28.05.2018	Wisentgehege Springe
Dekan	Reference	L	M	09.07.2018	Wisentgehege Springe
Dena	Reference	L	F	23.04.2017	Wisentgehege Springe
Denise	Reference	L	F	19.08.2017	Wisentgehege Springe
Denus	Reference	L	M	06.05.2017	Wisentgehege Springe
Eggehexe II	Reference	LC	F	01.05.2017	Wisentgehege Springe
Elaika	Reference	LC	F	14.06.2018	Wisentgehege Springe
Elisabeth	Reference	LC	F	10.05.2016	Wisentgehege Springe
Ella	Reference	LC	F	28.05.2017	Wisentgehege Springe
Elwira	Reference	LC	F	28.05.2018	Wisentgehege Springe
Hogir	Reference	LC	M	02.09.2017	Wisentgehege Springe
Spakko	Reference	LC	M	27.06.2018	Wisentgehege Springe
Spatz	Reference	LC	M	27.08.2018	Wisentgehege Springe
Spectra	Reference	LC	F	02.06.2017	Wisentgehege Springe
Spendera	Reference	LC	F	12.05.2018	Wisentgehege Springe
Spilla	Reference	LC	F	22.06.2017	Wisentgehege Springe
Spresa	Reference	LC	F	15.05.2017	Wisentgehege Springe
Tábor	Reference	L	M	07.11.2017	Tábor ZOO

**Table A2:** Primer sequences of selected regions surrounding potentially longevity-associated SNPs.

Gene	Forward primer sequence	Reverse primer sequence
BCKDHB	TGGCAGTGGGTACTCCTAGA	TGCCAGTAAATTGGGGGTGG
FER1L6	GGAATTCTACTGCTGTTGGCT	GTCCATTTGCCTGCACTGTC
SERPINI2	TGCCCAGATCTTAACCAGTGT	TCTTTCCGTTGAACCTACTATGA
XKR4	CCAGCCCATTCCTACCACTG	TCCCTCTCCCTGAGAAAGCA

**Table A3:** Results of sequencing of SNP positions in selected genes. X = not succesfuly sequenced.

Name	Group	Genotype BCKDHB	Genotype XKR4	Genotype FER1L6	Genotype SERPINI2
Bärnburger	Long-lived	X	AA	TC	TT
Baron	Long-lived	TT	AA	TC	TT
Bransk	Long-lived	TG	GG	TT	GG
Donna	Long-lived	TT	AA	CC	GT
Elvis	Long-lived	GG	X	CC	X
Erika	Long-lived	X	X	TT	TT
Gama	Long-lived	TT	AA	TC	TT
Indi	Long-lived	X	X	TT	X
Izafatty	Long-lived	TT	AG	TC	TT
Izvara	Long-lived	TG	AA	TC	GG
Maika	Long-lived	TT	GG	TT	GT
Nina	Long-lived	TT	AA	CC	TT
NL-25*	Long-lived	GG	AA	X	X
Plucik	Long-lived	TG	AA	CC	TT
Postójka	Long-lived	X	X	CC	X
Pristina	Long-lived	TT	AG	TC	TT
Raisa	Long-lived	TT	GG	TT	GT
Rimpsa	Long-lived	TG	AA	TC	TT
Sphinx	Long-lived	TG	AG	TC	X
Splunkunyr	Long-lived	X	X	CC	X
Thira	Long-lived	TT	AA	TC	GG
Zwursel	Long-lived	TG	AA	X	GT
Curt	Reference	GG	X	X	TT
Deisterix	Reference	TG	AA	CC	TT
Dekan	Reference	GG	AA	CC	X
Dena	Reference	GG	X	X	TT
Denise	Reference	GG	AA	CC	TT
Denus	Reference	TG	AA	CC	TT
Eggehexe II	Reference	X	X	TC	X
Elaika	Reference	TT	AA	TC	X
Elisabeth	Reference	X	AA	CC	TT
Ella	Reference	GG	AA	CC	TT
Elwira	Reference	TG	AA	TC	X
Hogir	Reference	TT	AA	CC	TT
Spakko	Reference	X	X	CC	X
Spatz	Reference	X	X	CC	X
Spectra	Reference	GG	AA	CC	TT
Spendera	Reference	X	X	X	TT
Spresa	Reference	X	X	CC	TT
Tábor	Reference	GG	AA	CC	TT

## Appendix A

Table A1, Table A2, Fig. A1, Fig. A2, Table A3, Fig. A3
